# Hepatitis B antibody levels after different doses of hepatitis B vaccination: a retrospective study based on hospitalized children

**DOI:** 10.1017/S0950268823001747

**Published:** 2023-10-26

**Authors:** Yu Chai, Jihai Tang, Yin Su, Kun Xuan, Lili Xu, Jiayan Hao, Zhijian Lu, BinBing Wang, Xia Chen, Xianwei Luo, Jiali He, Lijuan Zhu

**Affiliations:** 1 Anhui Provincial Center for Disease Control and Prevention, Hefei, China; 2 Children’s Hospital of Fudan University Anhui Hospital, Heifei, China

**Keywords:** hepatitis B surface antibody, hepatitis B vaccine, hospitalized patients, positivity rate, geometric mean concentration

## Abstract

Many studies have investigated the positivity rate of hepatitis B surface antibody (HBsAb) after hepatitis B vaccine (HepB) immunization. However, the antibody level, assessed monthly or at more frequent intervals after each of the three doses, particularly within the first year after birth, has not been previously reported. To elucidate the level of antibody formation at various times after vaccination, the current study used the available detection data of HBsAb in hospitalized children to analyze the HBsAb level after immunization combined with their vaccination history. Both the positivity rate and geometric mean concentration (GMC) increased sequentially with immunization doses, reaching their peaks earlier after the third dose than after the first two doses, and the rate of HBsAb positivity was able to reach 100% between 11 and 90 days after completing the three doses of HepB. Within one year after receiving the three doses, the antibody positivity rate and GMC were maintained above 90% and 100 mIU/mL, respectively, and subsequently steadily declined, reaching the lowest value in the 9th and 10th years. The current findings reveal, in more detail, the level of antibody formation at different times following each dose of HepB in hospitalized children, particularly in the age group up to one year after vaccination. For the subjects of this study, we prefer to believe that the proportion of HBsAb non-response should be less than 5% after full immunization with HepB, provided that the appropriate time for blood collection is chosen.

## Introduction

Hepatitis B is a disease caused by the hepatitis B virus (HBV), which is mainly associated with liver inflammatory lesions and can result in multiple organ damage. The World Health Organization (WHO) has estimated that 296 million people were living with chronic hepatitis B infection in 2019, with 1.5 million new infections each year. In addition, the number of related deaths in 2019 was 820,000, mainly caused by HBV-induced liver cirrhosis and liver cancer [1]. Nowadays, there is no method that can cure chronic hepatitis B infection completely, but the hepatitis B vaccine (HepB) is the most effective method to prevent HBV infection [2]. In 1992, HepB was included in the planned immunization management in China, and it was further included in the Childhood Immunization Programme to implement free vaccination for newly born children in 2002, with efforts being made to improve the whole-course vaccination rate of children and the timely vaccination rate for the HepB birth dose (HepB-BD).

At present, numerous studies have focussed on epidemiological investigation of the positivity rate of hepatitis B surface antibody (HBsAb) in people over 1 year old [3–9]. However, the antibody level, assessed monthly or at more frequent intervals after each of the three doses, has not been previously reported. To elucidate the level of antibody formation at various times after injection, the current study used the detection data of HBsAb and hepatitis B surface antigen (HBsAg) of hospitalized children to analyze the HBsAb level after immunization, with reference to their vaccination history.

## Methods

### Subjects

**Figure 1.**
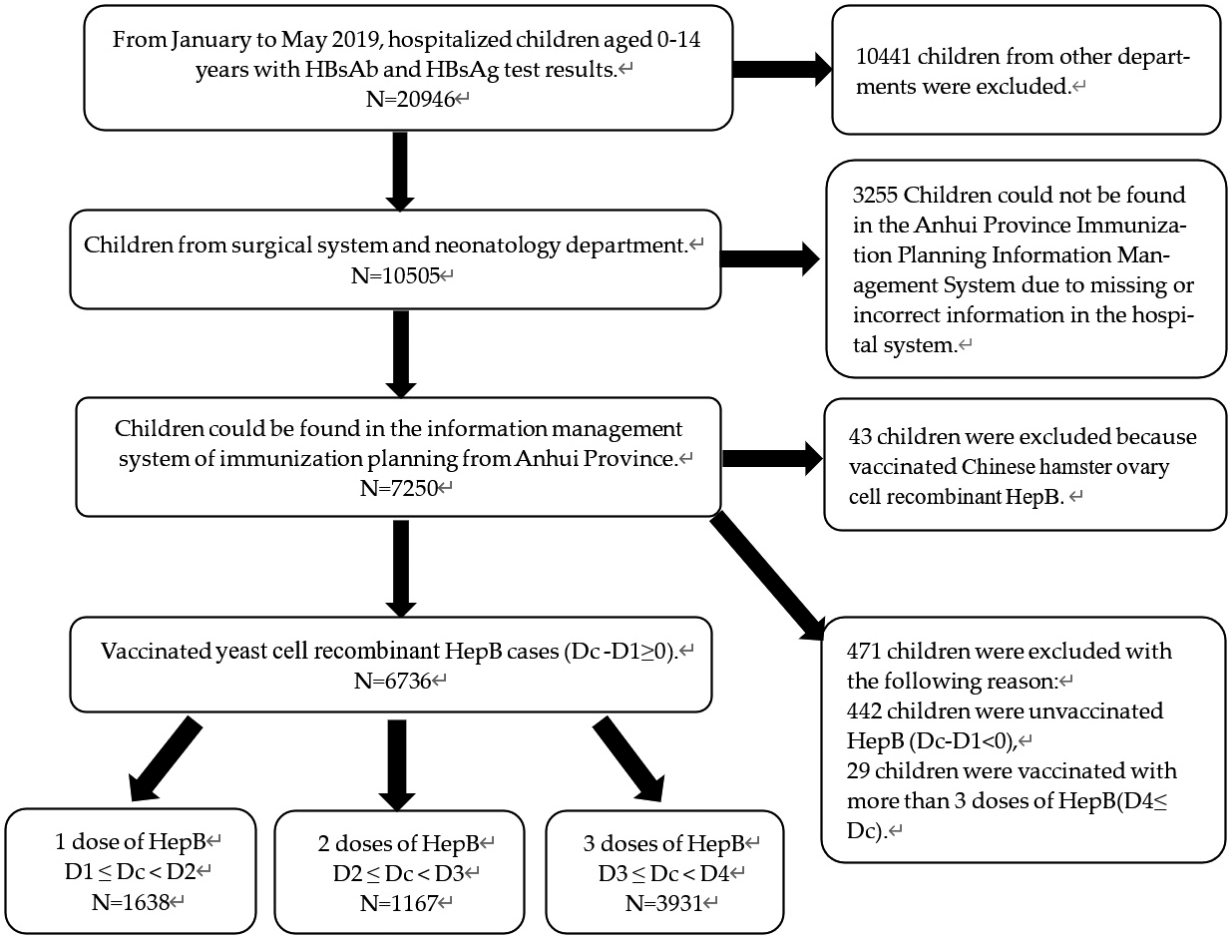
Flow diagram of selection and classification of subjects. Dc: Date of blood collection; Db: Date of birth; D1: Date of the first dose of HepB; D2: Date of the second dose of HepB; D3: Date of the third dose of HepB; D4: Date of the fourth dose of HepB. 1 dose HepB: The group only vaccinated one-dose HepB; 2 doses HepB: Completed two doses of HepB vaccination; 3 doses HepB: Completed three doses of HepB vaccination.

Retrospective collection of serological data from children hospitalized in Anhui Provincial Children’s Hospital from January to May 2019, a total of 20,946 hospitalized children aged 0–14 years were tested for HBsAb and HBsAg in Anhui Provincial Children’s Hospital, of which 7,250 hospitalized children in the surgical system (general surgery, ophthalmology, otolaryngology, orthopaedic surgery, urology, and stomatology) and neonatal department were selected, and their vaccination information was collected from the Anhui immunization planning information management system. After excluding 43 children who received the Chinese hamster ovary cell recombinant HepB, 442 who were unvaccinated against HepB, and 29 who were vaccinated with more than 3 doses of HepB, these 6,736 children vaccinated Yeast cell recombinant HepB (not the same brand) were divided into one-dose, two-dose, or three-dose groups according to their hepatitis B vaccination status (See Figure 1).

### Sample collection and detection

Children’s fasting peripheral venous blood samples of 2–3 mL were drawn, centrifuged at 4000 rpm for 15 min, and then the supernatant was collected. HBsAg and HBsAb markers were detected in serum using the CL-2000i automated chemiluminescent immunoassay analyzer according to the instructions for the HBsAg and HBsAb detection kits.

### Reagents and instruments

A CL-2000i automatic chemiluminescent immunoassay analyzer, HBsAg and HBsAb detection kits, and HBsAg and HBsAb quality control samples were all purchased from China Mindray Medical Co., Ltd.

### Result interpretation standard

Referring to the manuals of these detection kits, an HBsAg concentration of ≥0.08 IU/mL was regarded as positive; otherwise, it was negative. An HBsAb concentration of ≥10.0 mIU/mL was regarded as positive; otherwise, it was negative. The results of both the HBsAg and HBsAb quality control samples were within the quality control range.

### Statistical analysis

Excel 2016 and SPSS 13.0 software were used for statistical analysis. The HBsAb concentration is described by the geometric mean concentration (GMC) and its 95% confidence intervals (CI). HBsAb and HBsAg levels were analyzed within 1 year after one, two, and three doses of HepB vaccination, respectively. Moreover, the antibody levels at different times within 14 years after vaccination in children who were fully vaccinated were also analyzed.

## Results

### Demographic characteristics of the subjects

A total of 6,736 subjects were included in this study, of which 4,418 were male and 2,318 were female. The overall positivity rate of HBsAb and HbsAg were 76.02% (5,121/6736) and 0.06% (4/6736). Most of the children were from general surgery, urology, and neonatal department. The HepB vaccination procedure for children is 10 μg three doses of yeast cell recombinant HepB at zero, one, and six months. At the time of blood collection, 1,638, 1,167, and 3,931 children had received one dose, two doses, and a full dose of HepB, respectively (See Table 1).

**Table 1.** General characteristics and vaccination history of the subjects

Characteristic	One dose of HepB	Two doses of HepB	Three doses of HepB
Sex, *N*(%)			
Male	989 (60.38)	745 (63.84)	2,684 (68.28)
Female	649 (39.62)	422 (36.17)	1,247 (31.72)
Age of blood collection (day), M(IQR)	6.0 (15.0, 28.0)	135.0(86.0, 191.5)	701.0 (449.0, 1,740.0)
Age of vaccination (day), M(IQR)	0 (0, 1.0)	35.0 (32.0, 47.0)	202.0 (189.0, 222.0)
Source, *N*(%)			
General surgery	214 (13.06)	823 (70.52)	1,943 (49.43)
Ophthalmology	4 (0.24)	61 (5.23)	186 (4.73)
Otolaryngology	0 (0)	4 (0.34)	186 (4.73)
Orthopaedic surgery	6 (0.37)	41 (3.51)	487 (12.39)
Urology	26 (1.59)	140 (12.00)	932 (23.71)
Stomatology	0(0)	10 (0.86)	127 (3.23)
Neonatal department	1,388 (84.74)	88 (7.54)	70 (1.78)
Time (day), *N*(%)	Dc-D1	Dc-D2	Dc-D3
1-	678 (41.39)	47 (4.03)	43 (1.09)
11-	351 (21.43)	80 (6.86)	33 (0.84)
21-	291 (17.77)	83 (7.11)	40 (1.02)
31-	201 (12.27)	199 (17.05)	122 (3.10)
61-	41 (2.50)	173 (14.82)	137 (3.49)
91-	35 (2.14)	427 (36.59)	385 (9.79)
181-	21 (1.28)	104 (8.91)	835 (21.24)
366-	20 (1.22)	54 (4.63)	2,336 (59.43)
Total	1,638 (100)	1,167 (100)	3,931 (100)

Abbreviations: Dc, date of blood collection; D1, date of the first dose of HepB; D2, date of the second dose of HepB; D3: Date of the third dose of HepB.

### HBsAb levels in the one-dose HepB group

**Figure 2.**
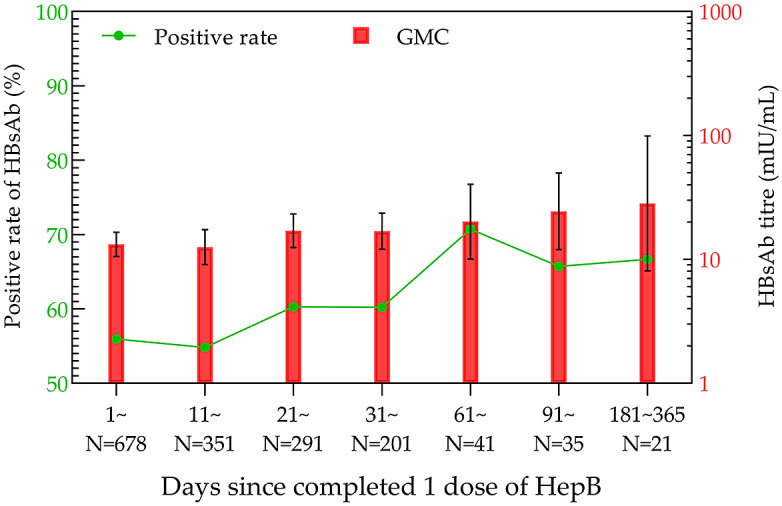
HBsAb levels in the one-dose HepB group within one year.

Within one year after the first HepB vaccination, the positivity rate volatility rose, with the highest rate of 70.73% in the 61–90-day group. Meanwhile, GMC showed a gentle increasing trend, and the highest GMC was 28.24 mIU/mL (95% CI: 8.07–98.76 mIU/mL) in the 181–365-day group (Figure 2).

### HBsAb levels in the two-dose HepB group

**Figure 3.**
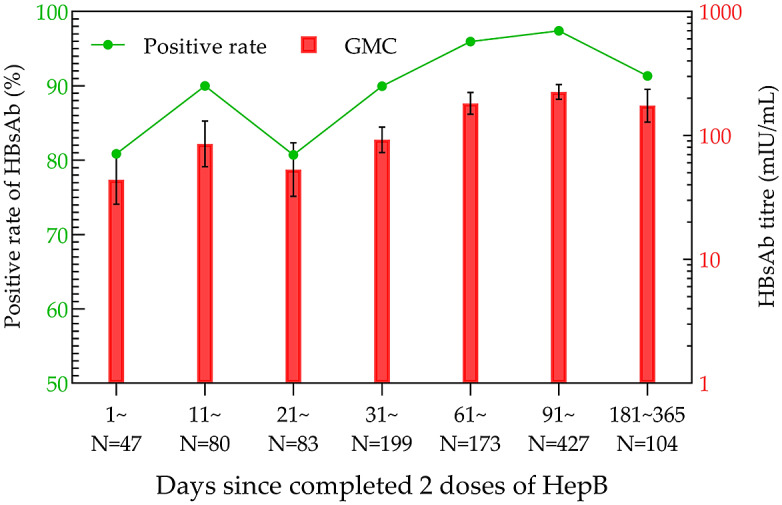
HBsAb levels in the two-dose HepB group within one year.

Although there were some variations in the data for the 21–30- and 181–365-day groups, there was an increasing trend in the overall positivity rates and GMC after the second dose of HepB. The positivity rate and GMC of HBsAb were the highest in the 91–180-day group, which were 97.42% and 224.11 mIU/mL (95% CI: 195.50–256.90 mIU/mL), respectively (Figure 3).

### HBsAb levels in the three-dose HepB group

**Figure 4.**
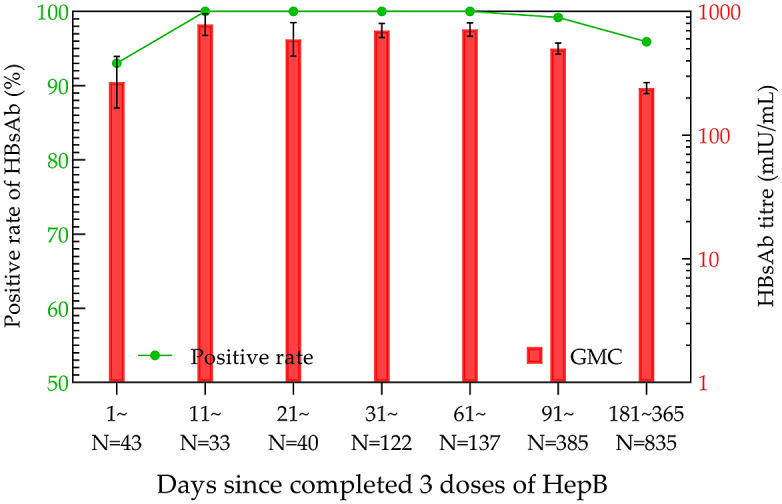
HBsAb levels in the three-dose HepB group within one year.

**Figure 5.**
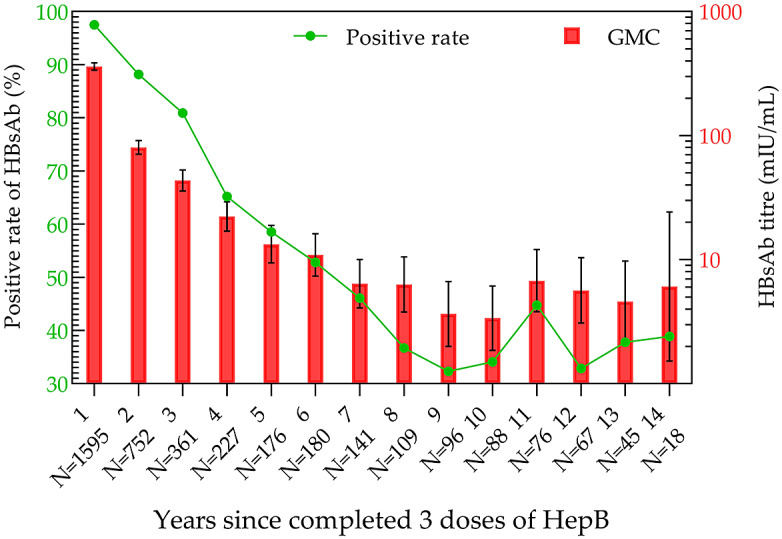
HBsAb levels during the 14 years in the three-dose HepB group.

After the completion of full immunization, the positivity rate and GMC of HBsAb were the highest in the 11–20-day group, which were 100% and 784.15 mIU/mL (95% CI: 640.49–960.03 mIU/mL), respectively (Figure 4). Then, the analysis of antibody levels in 3931 children within 14 years after three doses of immunization demonstrated that the positivity rate and GMC of HBsAb decreased with time. The lowest positivity rate (32.29%) and GMC (3.37 mIU/mL; 95% CI: 1.85–6.13 mIU/mL) were observed at years 9 and 10 post-vaccination, respectively. After that, the positivity rate and GMC were maintained at 30%–45% and 3–7 mIU/mL until the 14th year, respectively (Figure 5).

### HBsAb level within one year after one, two, and three doses of HepB

The positivity rates and GMC of HBsAb were higher with increasing doses of HepB, and their time to peak was shorter with each additional dose. The positivity rate and GMC of HBsAb remained above 90% and 100 mIU/mL, respectively, within one year after the completion of three vaccination doses.

## Discussion

The pattern of antibody formation after vaccine immunization has been described in many papers and textbooks, but it was difficult to observe the precise antibody level after one, two, and three doses by continuous blood collection among young children, especially within one year of birth. This was partly due to the difficulty of collecting blood multiple times from children in the younger age groups, the obstacle of obtaining support from children and their parents, and the high cost of organizing investigations and blood collection. Therefore, firstly, most studies collected serum at 1–3 time points after immunization [10–12]. Secondly, these time points cover a wide span of age groups, with few data available up to one year after vaccination [13]. Thirdly, a few studies collected blood samples in certain months of the 1-year-old age group, but the sample size per group was relatively small, with a maximum of 30 samples per group in the study by Jilg et al. [14]. Therefore, previous descriptions of antibody levels after three doses of HepB vaccination were not detailed and adequate. Regarding these, the current study used the available detection data of HBsAb and hepatitis B surface antigen (HBsAg) in hospitalized children (including a large number up to one year after vaccination) to analyze the HBsAb level after immunization combined with their vaccination history. With the data now available, our study aims to evaluate the hepatitis B antibody level after 1, 2, and 3 doses of vaccination. This will serve as a foundation for a subsequent investigation into the dynamic evolution of antibodies.

The results of the current study demonstrated that the highest positivity rates of HBsAb appeared at 61–90 days after HepB-BD immunization (70.73%), 91–120 days after the second dose of HepB immunization (97.42%), and 11–20 days after the third injection of HepB (100%), respectively. Correspondingly, the highest GMC values after each dose of HepB vaccination showed at 181–365 days (28.24 mIU/mL; 95% CI: 8.07–98.76 mIU/mL), 91–180 days (224.11 mIU/mL, 95% CI: 195.50–256.90 mIU/mL), and 11–20 days (784.15 mIU/mL; 95% CI: 640.49–960.03 mIU/mL). Both the positivity rate and GMC increased sequentially with the immunization dose, reaching their peaks earlier after the third dose than after the first two doses.

For the evaluation of immunization efficacy after HepB vaccination, the WHO position paper (2017 version) [15] stated that a positive HBsAb (≥10 mIU/mL) in the blood, collected one to two months after the completion of full HepB immunization (one to three months after immunization was propounded in *Vaccines* [16]), indicated long-term effective protection against HBV. Many studies have reported that approximately 5% of the vaccinees fail to produce effective protective antibodies (HBsAb <10 mIU/mL) after HepB vaccination. In contrast, all 332 cases tested positive for HBsAb in blood collected 11–90 days after three full doses of immunization in our study, which was higher than that reported in previous studies. These studies could be divided into the following categories. In the first category, specimens were collected more than three months after immunization [17–19], or even two years after immunization [20]. When our study followed their blood collection time, the results were also similar. The second category was that the study population was a high-risk group for hepatitis B, such as close relatives of HBV carriers [13] and newborns of HBsAg-positive mothers [19, 21]. Then, the third included studies in which the subjects were based on or included adolescent populations [22, 23]. Previous studies have illustrated the fact that the peak of HBsAb formation in older children or teenagers is slightly later than that in neonates [24, 25], thus being more likely to lead to undetectable HBsAb. The last category included studies aiming at different immunization procedures. Jilg et al. [14], Goldfarb et al. [26], Sintusek et al. [27], and Greenberg et al. [28] studied the difference in the HBsAb positivity rate according to varying vaccination times (0, 1, 6; 0, 1, 12; 2, 4, 6 months) and varying dosages (5, 10, and 20 μg immunization). Taking the above factors into account, it could be reasonably explained that the positivity rate of HBsAb in the current study was higher than that in previous studies. Therefore, we prefer to believe that the proportion of HBsAb non-response should be less than 5% after full immunization with HepB in a normal population, provided that the appropriate time for blood collection is chosen.

For the subjects of this study, it was more appropriate to collect blood 11–90 days after the three doses of HepB immunization to detect HBsAb. The present timeline was more precise than the one- to two- and one- to three-month time frames indicated in the WHO position paper (2017 version) and *Vaccines.*

In terms of antibody duration, the positivity rate and GMC of HBsAb could be maintained at above 90% and 100 mIU/mL after three doses of HepB immunization, respectively, within one year. The positivity rate and GMC fell below 50% and 10 mIU/mL by seven years post-vaccination and reached the lowest (32.29% and 3.37 mIU/mL) by years 9 and 10. The highest and lowest positivity rates of HBsAb in the 2014 Anhui Province Hepatitis B Serological Survey were also found in the one-year-old group (91.89%) and the 9–10-year-old group (39.13%) [29]. The two results were consistent. However, the 2014 survey only conducted qualitative testing, so the GMC could not be calculated. Furthermore, the study subjects were between 1 and 29 years old, and children younger than one year old were not included.

Although this was the first study to specifically explore the antibody levels 0–14 years after HepB immunization in hospitalized children, there were some limitations to this work. First, the data are not longitudinal, and there is individual heterogeneity in antibody levels after each of the three doses; therefore, they can only be used to provide a reference for the dynamic change of antibody levels after immunization. Finally, there was selection bias when compared to the normal population since the subjects of this study were inpatients (unhealthy children). In the selection of the study population, we excluded cases from the department that were prone to hepatitis B patient visits; therefore, the number of hepatitis B virus carriers and infections with no or low response to the vaccine was reduced. As a result, the HBsAg positivity rate of the included subjects was closer to that of the normal population. The results of this study showed that the HBsAg positivity rate was 0.06% (4/6736), consistent with the decreasing trend of HBsAg in several serological surveys of hepatitis B in Anhui Province (the positivity rate of HBsAg in children aged 1–14 years in 2006 was 1.49% (20/1340) [30]and in 2014 was 0% (0/851) [29]. And among children <15 years of age, HBsAg prevalence declined from 10.5% to 0.8% in China, during 1992–2014 [31]), expecting that the results of the analysis would have some reference values for the normal population, although this is the biggest limitation of this study.

## Data Availability

The data presented in this study are available upon request from the corresponding author. The data are not publicly available due to them containing private information about individuals.

## References

[1] WHO (2022). World Health Organization Fact Sheet-Hepatitis B.

[2] Chinese Society of Infectious Diseases CMA, Chinese Society of Hepatology CMA (2019). Guidelines for the prevention and treatment of chronic hepatitis B (version 2019).

[3] Fuzhen W, et al. (2017). Comparative analyze on hepatitis B seroepidemiological surveys among population aged 1-29 years in different epidemic regions of China in 1992 and 2014. Chinese Journal of Preventive Medicine 51, 462–468. 28592086 10.3760/cma.j.issn.0253-9624.2017.06.002

[4] Lopez-Gatell H, et al. (2019). Hepatitis B seroprevalence in 10-25-year-olds in Mexico - the 2012 national health and nutrition survey (ENSANUT) results. Human Vaccines and Immunotherapeutics 15, 433–439. 10.1080/21645515.2018.1533617 30380981 PMC6422518

[5] Verso MG, et al. (2019). Immunization against Hepatitis B Surface Antigen (HBsAg) in a cohort of nursing students two decades after vaccination: surprising feedback. Vaccines (Basel) 8, 1. 10.3390/vaccines8010001 31861551 PMC7157657

[6] Janzen J, et al. (1978). Epidemiology of hepatitis B surface antigen (HBsAg) and antibody to HBsAg in hospital personnel. Journal of Infectious Diseases 137, 261–265. 10.1093/infdis/137.3.261 632624

[7] King H, et al. (2020). Trends in prevalence of protective levels of Hepatitis B surface antibody among adults aged 18-49 years with risk factors for Hepatitis B virus infection-United States, 2003-2014. Clinical Infectious Diseases 70, 1907–1915. 10.1093/cid/ciz537 31228240 PMC7440671

[8] Lee KH, et al. (2017). Changes in hepatitis B virus antibody titers over time among children: a single center study from 2012 to 2015 in an urban of South Korea. Biomed Central Pediatrics 17, 164. 10.1186/s12887-017-0924-7 28705230 PMC5512724

[9] Zanella B, et al. (2020). Hepatitis B seroprevalence in the pediatric and adolescent population of florence (Italy): an update 27 years after the implementation of universal vaccination. Vaccines (Basel) 8, 156. 10.3390/vaccines8020156 32235670 PMC7348992

[10] Ballesteros-Trujillo A, et al. (2001). Response to hepatitis B vaccine in preterm infants: four-dose schedule. American Journal of Perinatology 18, 379–385. 10.1055/s-2001-18695 11731891

[11] Watanaveeradej V, et al. (2002). Antibody response to hepatitis B vaccine in infants of HIV-positive mothers. International Journal of Infectious Diseases 6, 240–241. 10.1016/s1201-9712(02)90120-7 12718844

[12] Milne A, et al. (2002). Field evaluation of the efficacy and immunogenicity of recombinant hepatitis B vaccine without HBIG in newborn Vietnamese infants. Journal of Medical Virology 67, 327–333. 10.1002/jmv.10071 12116022

[13] Yuen MF, et al. (2004). 18-year follow-up study of a prospective randomized trial of hepatitis B vaccinations without booster doses in children. Clinical Gastroenterology and Hepatology 2, 941–945. 10.1016/s1542-3565(04)00384-2 15476159

[14] Jilg W, Schmidt M, Deinhardt F (1989). Vaccination against hepatitis B: comparison of three different vaccination schedules. Journal of Infectious Diseases 160, 766–769. 10.1093/infdis/160.5.766 2530289

[15] WHO (2017). Hepatitis B vaccines: WHO position paper.

[16] Plotkin SA, Orenstein WA, Offit PA (2012). Vaccines, 6th Edn. Netherlands: Elsevier.

[17] Hui Z, Fu-zhen W, Yuan-sheng C (2007). Infants non-and low-response after recombinant yeast derived Hepatitis B vaccinated and influencing factors analysis. Chinese Journal of Vaccines and Immunization 13, 303–305.

[18] Cleveland JL, et al. (1994). Factors associated with hepatitis B vaccine response among dentists. Journal of Dental Research 73, 1029–1035. 10.1177/00220345940730050301 8006228

[19] Safadi R, et al. (2021). Efficacy of birth dose vaccination in preventing mother-to-child transmission of hepatitis B: a randomized controlled trial comparing Engerix-B and Sci-B-Vac. Vaccines (Basel) 9. 10.3390/vaccines9040331 PMC8066861 33915943

[20] Nani X, et al. (2021). Two-year hepatitis B antibody sero-prevalence following sequential primary vaccination with domestic and imported recombinant yeast-based hepatitis B vaccines. Chinese Journal of Vaccines and Immunization 27, 501–503+508. 10.19914/j.CJVI.2021086

[21] Zou H, et al. (2011). Protective effect of hepatitis B vaccine combined with two-dose hepatitis B immunoglobulin on infants born to HBsAg-positive mothers. Public Library of Science One 6, e26748. 10.1371/journal.pone.0026748 22053208 PMC3203892

[22] Catania G, Di Ciommo V, Concato C (1996). Vaccination against hepatitis B virus in children and adolescents in a pediatric hospital. Recenti Progressi in Medicina 87, 271–274. 8766952

[23] Stevens CE, et al. (1987). Yeast-recombinant hepatitis B vaccine. Efficacy with hepatitis B immune globulin in prevention of perinatal hepatitis B virus transmission. The Journal of the American Medical Association 257, 2612–2616. 10.1001/jama.257.19.2612 2952812

[24] Honorati MC, et al. (1999). A mathematical model predicting anti-hepatitis B virus surface antigen (HBs) decay after vaccination against hepatitis B. Clinical and Experimental Immunology 116, 121–126. 10.1046/j.1365-2249.1999.00866.x 10209515 PMC1905208

[25] Hwang LY, et al. (1983). Immunogenicity of HBV vaccine in healthy Chinese children. Vaccine 1, 10–12. 10.1016/0264-410x(83)90005-1 6241767

[26] Goldfarb J, et al. (1996). Comparison study of the immunogenicity and safety of 5- and 10-microgram dosages of a recombinant hepatitis B vaccine in healthy infants. Pediatric Infectious Disease Journal 15, 764–767. 10.1097/00006454-199609000-00004 8878217

[27] Sintusek P, et al. (2022). Safety and immunogenicity of standard and double doses of hepatitis B vaccine in children after liver transplantation: an open-label, randomised controlled trial. Vaccines (Basel) 10. 10.3390/vaccines10010092 PMC8778427 35062752

[28] Greenberg DP, et al. (1996). Comparative safety and immunogenicity of two recombinant hepatitis B vaccines given to infants at two, four and six months of age. Pediatric Infectious Disease Journal 15, 590–596. 10.1097/00006454-199607000-00006 8823852

[29] Ying S, Jihai T, Yu C (2017). Sero epidemiological survey of hepatitis B virus among people aged 1 -29 in Anhui Province, 2014. Modern Preventive Medicine 44, 1553–1556+1560.

[30] Yu C, et al. (2018). Acute hepatitis B in Anhui province, 2009 to 2016. Chinese Journal of Vaccines and Immunization 24, 280–284.

[31] Cui F, et al. (2017). Prevention of chronic hepatitis B after 3 decades of escalating vaccination policy, China. Emerging Infectious Diseases 23, 765–772. 10.3201/eid2305.161477 28418296 PMC5403029

